# Exploring neighborhood inequality in female breast cancer incidence in Tehran using Bayesian spatial models and a spatial scan statistic

**DOI:** 10.4178/epih.e2017021

**Published:** 2017-05-17

**Authors:** Erfan Ayubi, Mohammad Ali Mansournia, Ali Ghanbari Motlagh, Alireza Mosavi-Jarrahi, Ali Hosseini, Kamran Yazdani

**Affiliations:** 1 Department of Epidemiology and Biostatistics, School of Public Health, Tehran University of Medical Sciences, Tehran, Iran; 2 Department of Radiotherapy, Shahid Beheshti University of Medical Sciences, Tehran, Iran; 3 Department of Health and Community Medicine, Shahid Beheshti University of Medical Sciences, Tehran, Iran; 4 Department of Geography and Urban Planning, University of Tehran, Tehran, Iran

**Keywords:** Breast neoplasms, Spatial analysis, Health status disparities, Iran

## Abstract

**OBJECTIVES:**

The aim of this study was to explore the spatial pattern of female breast cancer (BC) incidence at the neighborhood level in Tehran, Iran.

**METHODS:**

The present study included all registered incident cases of female BC from March 2008 to March 2011. The raw standardized incidence ratio (SIR) of BC for each neighborhood was estimated by comparing observed cases relative to expected cases. The estimated raw SIRs were smoothed by a Besag, York, and Mollie spatial model and the spatial empirical Bayesian method. The purely spatial scan statistic was used to identify spatial clusters.

**RESULTS:**

There were 4,175 incident BC cases in the study area from 2008 to 2011, of which 3,080 were successfully geocoded to the neighborhood level. Higher than expected rates of BC were found in neighborhoods located in northern and central Tehran, whereas lower rates appeared in southern areas. The most likely cluster of higher than expected BC incidence involved neighborhoods in districts 3 and 6, with an observed-to-expected ratio of 3.92 (p<0.001), whereas the most likely cluster of lower than expected rates involved neighborhoods in districts 17, 18, and 19, with an observed-to-expected ratio of 0.05 (p<0.001).

**CONCLUSIONS:**

Neighborhood-level inequality in the incidence of BC exists in Tehran. These findings can serve as a basis for resource allocation and preventive strategies in at-risk areas.

## INTRODUCTION

Statistics have shown that the incidence rate of breast cancer (BC) is 24 per 100,000 in women in Iran [1]. The annual number of new BC cases is projected to increase from 5,000 in 2000 to 15,000 in 2030 [2]. BC has a poor prognosis in Iran; it is the third leading cause of death from cancers, accounting for 16% of cancer deaths [3]. Most BC patients are diagnosed at advanced stages in Iran [3].

The incidence and mortality of BC have been attributed to many individual-level risk factors [4-9]. Regardless of these risk factors, it has been found that the incidence and mortality of BC are associated with place-based and area-based risk factors [9,10]. While many studies in other countries have considered the spatial patterns of BC at the census tract or zip code level [9,11,12], spatial patterns in the incidence of BC have been studied only at the provincial level in Iran [13-15]. For example, the overall incidence of BC in the population living in Tehran province was 31.5 per 100,000, which is greater than the rates observed in other provinces [16]. Therefore, studies should focus on identifying spatial patterns of BC incidence on finer geographic scales to understand health needs and to optimize health care allocation [9-11].

When studying the spatial patterns of disease on a finer geographic scale, however, some challenges must be considered. Estimated rates and observed associations can involve a degree of bias due to spatial autocorrelation, population size heterogeneity, and small-area effects [17]. Two methods, empirical spatial Bayesian smoothing and the Besag, York, and Mollie (BYM) spatial model, have been used to offset these challenges by considering spatial autocorrelation and spatial heterogeneity among geographic units [11,18,19].

With these issues in mind, our objectives in this study were (1) to estimate the smoothed standardized incidence ratio (SIR) among neighborhoods in Tehran, and (2) to identify clusters of higher or lower than expected incidence of female BC in Tehran.

## MATERIALS AND METHODS

### Study area

A retrospective study design was used in Tehran, the capital of Iran. This city has 22 districts. The geographical units of the study were 374 neighborhoods in the city of Tehran.

### Data sources

Information about incident cases of female BC in Tehran from March 2008 to March 2011 was obtained from the cancer registry of the Ministry of Health of Iran. Patients’ street of residence was geocoded to the neighborhood. The population of women aged 15 and over in each neighborhood was obtained from the national census of 2006 and 2011.

### Statistical analysis

#### Raw standardized incidence ratio

The number of the observed events in each neighborhood follows a Poisson distribution,

(1) $$O_{i}\sim Poisson(E_{i}\theta_{i})$$

where *O_i_*, *E_i_*, and *θ_i_* are the observed number, of casesnumber, the expected number of cases, and the relative risk for neighborhood *i*, respectively. The number of expected events is calculated as follows:

(2) $$E_{i}=n_{i}\left(\frac{\sum_{iyi}}{\sum_{ini}}\right),\;i=1,2,\;....\;I$$

where *n_i_* is the number of women aged 15 and over in neighborhood *i* $\left(\frac{population\;in\;2011+population\;in\;2006}{2}\right)$, and *y_i_* is the observed number of events in the neighborhood *i*. The SIR can be calculated by as the observed observed-to to-expected ratio.

### Besag, York, and Mollie model

Overdispersion or extra-Poisson variability is a challenge when the Poisson model is applied for the count data in a spatial analysis. Overdispersion occurs in the presence of spatial autocorrelation in the residual values. The concept of spatial autocorrelation refers to the idea that due to spatial components, the local estimates of disease risk for neighboring areas are assumed to be correlated. The effect of overdispersion due to spatial autocorrelation on the results is strong if the small-area problem is present [18].

To offset these challenges, hierarchical models such the BYM model have been introduced [18]. In the BYM model, unmeasured spatial factors are controlled for using suitable random effects, as shown in equation:

(3) $$\log(\theta_{i})=\alpha +u_{i}+v_{i}$$

where *α* is a the log-relative risk baseline, and *v_i_* and *u_i_* indicates random random-effects components regarding to spatial and non-spatial factors.

Spatial autocorrelation across neighborhoods (*v_i_*) is induced by the conditional autoregressive (CAR) model. The CAR model represents risk factors with spatial structures, so that specific risk estimates of a given area will tend to shrunk shrink toward a local mean. The CAR model within the BYM model is as follows:

where

(4) vivj, i≠j, τv2~N(v¯i, τi2)

(5) τi2=τv2∑jWij and vi =1∑jWij∑jvjwij

If areas *i* and *j* are neighbors of each other, the weight is equal to 1, and otherwise the weight is 0.

The random effect of *u_i_* represents risk factors with non-spatially structures, so such that that the specific risk estimate of a given area will tend to shrunk shrink toward a the global mean of the study area. This component in the BYM model is as follows:

(6) $$u_{i}\sim N(0,\tau_{u}^{2})$$

The parameters $\tau_{v}=\frac{1}{\delta_{v}^{2}}$ and $\tau_{u}=\frac{1}{\delta_{u}^{2}}$ are 2 precision parameters of the 2 aforementioned random effects. The proper distribution for *τ_v_* and *τ_u_* is the gamma distribution G(a, b) with expected value $\frac{a}{b}$ and variance $\frac{a}{b^{2}}$. In this study, based on previous studies designed to selec select the suitable gamma distribution [17,18,20], we used *a_v_*=0.5 and *b_v_*=0.005 for spatially structured random effects and *a_u_*=0.5 and *b_u_*=0.5 for non-spatially structured random effects.

We implemented a Markov-chain Monte Carlo (MCMC) simulation for estimating all parameters in the BYM model. The Gibbs sampler as a specific MCMC was used to produce random samples through the parameter space. The convergence of the model was evaluated by Brooks-Gelman-Rubin statistics [18]. This statistic method evaluates MCMC convergence by comparing the within-chain variance to the between-chain variance, with values close to 1 indicating the degree of convergence [18]. We ran the MCMC model with 100,000 iterations, ignoring the first 5,000 iterations as burn-in. Iterations started from overdispersed initial values on 2 parallel chains. OpenBUGS version 3.2.3 (http://www.openbugs.net/w/Downloads) was used to implement the BYM model.

### Spatial empirical Bayesian methods

Another available method for correcting bias in raw estimates of the SIR is spatial empirical Bayesian (SEB) methodsanalysis. The SEB method causes the rates in neighborhoods in areas without clear spatial patterns and in those in areas with obvious spatial patterns to be shrunk toward the global mean and local mean of the study area, respectively [21]. In this method, the posterior probability of *θ_i_* does depend on the data *O_i_* and *E_i_* from the other regions (*j*≠*i*). In other words, the parameters of the prior distribution are not fixed, and will beare estimated empirically and based on all available data. Smoothing raw SIRs with empirical bayes Bayesian methods was done using second-order queen weights in GeoDa.

### Detection and identification of breast cancer clusters

Neighborhood variation in the incidence of BC (regardless of staging), and in early and late stages at diagnosis were determined by the purely spatial scan statistic in a discrete Poisson model, using SaTScan version 9.4.2 (Harvard Medical School, Boston, MA, USA). The analysis requires the number of cases, population counts, and the geographical coordinates (longitude and latitude) for each location. The standard purely spatial scan statistic imposes a circular window (spatial cluster) on the map and it moves across the study area to compare the number of disease cases in a geographic area (θ_in_) with disease cases outside that area (θ_out_). Since the results of this analysis can be sensitive to model parameters, particularly window size, the maximum spatial cluster size is defined using the Gini coefficient [22]. It has been argued that the Gini coefficient is a very intuitive and systematic way to identify the best collection and non-overlapping of clusters [22].

The number of cases in each location was Poisson-distributed, so we applied the exponential model-based spatial scan statistic using SaTScan. The likelihood ratio statistic (LRS) of the Poisson distribution (under the test hypothesis; H_o_: θ_in_=θ_out_; H_a_: θ_in_ ≠ θ_out_) for a specific window is proportional to 1:

(7) $${\left(\frac{c}{E[c]}\right)}^{c}{\left(\frac{C-c}{C-E[c]}\right)}^{C}$$

where *C* is the total number of BC cases, *c* is the observed number of BC cases within a window, *E[c]* is the crude expected number of cases within the window under the null hypothesis, and *C−E[c]* is the expected number of cases outside the window.

The statistical significance of the detected clusters was evaluated using randomization testing or Monte Carlo hypothesis testing because the exact distribution of the LRS was unknown. Under the null hypothesis, a large number of random datasets was generated and the LRS value for each random dataset was computed. ndom dataset was computed. The Monte Carlo p-value of a window was computed as $\frac{R_{beat}+1}{R+1}$, where *R_beat_* is the number of random datasets with a LRS higher than the LRS in the real dataset and *R* is the total number of random datasets. A window shows statistical significance at *α*=0.05 when its LRS is higher than approximately 95% of the LRS values of the random dataset. The windows with the most statistically significant likelihood ratios were defined as the most likely, secondary, and tertiary clusters, respectively. The p-values of <0.05 using 999 permutations were considered to indicate statistical significance within the Moran index and spatial clusters. Sufficient statistical power was ensured by the use of 999 replications in the Monte Carlo simulation. All cartographic manipulations and displays were performed in ArcGIS version 10.3 (Esri, Redlands, CA, USA).

## RESULTS

**Figure 1.**
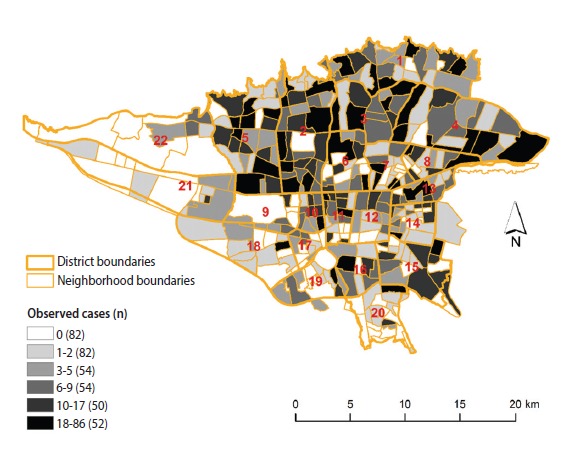
The number of observed female breast cancer cases across neighborhoods in Tehran, 2008-2011.

There were 4,175 incident BC cases in the study area from 2008 to 2011, and of them, 3,080 were successfully geocoded to the neighborhood. The number of BC cases ranged from 0 to 86 across neighborhoods in Tehran. The highest incidence of female BC was found in northern Tehran (Figure 1). Based on the Moran index, the null hypothesis of zero spatial autocorrelation was rejected for the number of BC (Moran index, 0.08; p<0.05).

### Spatial distribution of breast cancer

**Figure 2.**
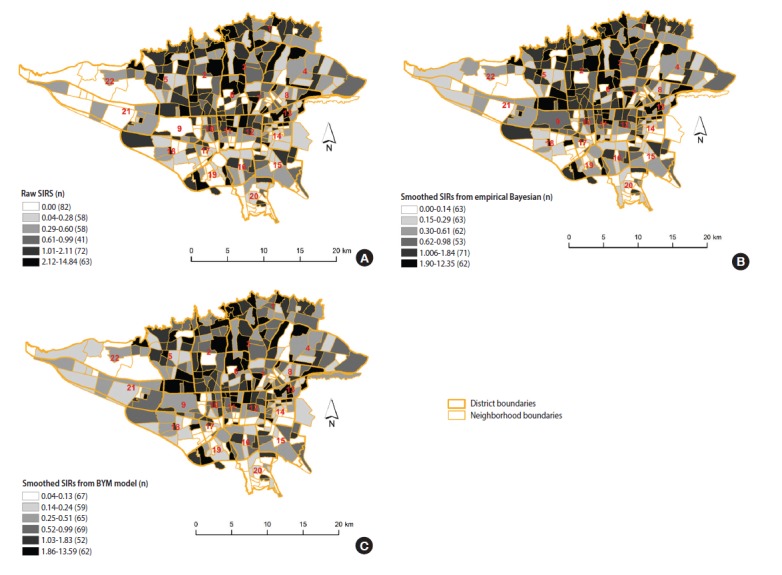
The estimated standardized incidence ratio (SIR) of female breast cancer incidence in Tehran, 2008-2011. (A) Raw SIRs, (B) using the spatial empirical Bayesian method, and (C) using the Besag, York and Mollie (BYM) spatial model.

The results of the three methods we used (raw SIR, the BYM model, and the SEB method) indicate neighborhood-level inequality in the incidence of female BC in Tehran. The neighborhoods with higher than expected incidence of BC were in districts 1, 2, 3, 5, 6 and 7, in northern and central Tehran. The neighborhoods with lower than expected rates were in districts 15, 16, 17, 18, 19, 20, 21, and 22, in southern and southwestern Tehran (Figure 2).

Figure 2A displays the estimated raw SIR of female BC in Tehran from 2008 to 2011. The median (interquartile range [IQR]) of female BC based on the raw SIR was 0.52 (1.33). The estimated raw SIR ranged from 0 to 14.84. In 82 neighborhoods, the raw SIR was 0 because no BC cases occurred in these neighborhoods; moreover, 37% of the neighborhoods had SIR values greater than 1. The smoothed SIRs using the SEB method are illustrated in Figure 2B. The median (IQR) of female BC based on the SEB method was 0.50 (1.13). As expected, there was a degree of shrinkage in the estimated SIR, such that in 2 neighborhoods the value of SIR was 0 and the range of SIRs was narrowed. The median (IQR) of female BC based on the BYM model was 0.60 (1.14), with no neighborhoods having a SIR of 0 and 30% of the neighborhoods having a SIR greater than 1 (Figure 2C).

### Spatial clusters of breast cancer

**Figure 3.**
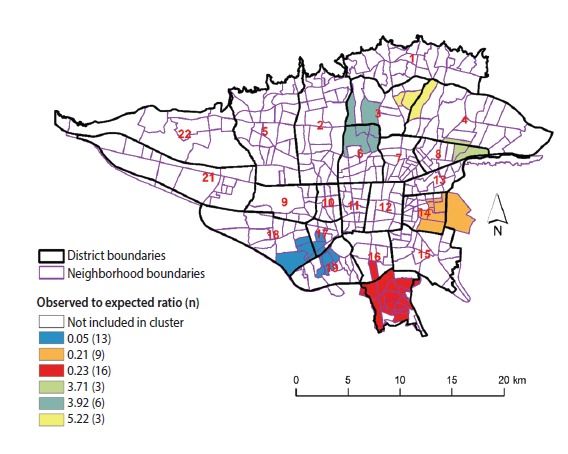
Spatial clusters of female breast cancer incidence in Tehran, 2008-2011.

Table 1 presents the characteristics of the most likely clusters of BC. Figure 3 displays the geographic pattern of the most likely clusters of BC. There was a statistical dispersion in the detected clusters of female BC incidence (Gini index, 0.47). The clusters with a higher than expected incidence of BC were found in the northern, northeastern, and central parts of the study area. Lower than expected incidence clusters of BC were found in the southern part of the study area. The most likely cluster of higher than expected BC incidence was located in areas near the center of Tehran, including neighborhoods in districts 3 and 6 with an observed-to-expected ratio of 3.92 (p<0.001), implying that the incidence of BC was 3.92 times greater within this cluster than in the rest of the study area. The most likely cluster of lower than expected BC incidence included the neighborhoods in districts 17, 18, and 19 with an observed-to-expected ratio of 0.05 (p<0.001), implying that the incidence of BC was 20 times lower within this cluster than in the rest of the study area.

## DISCUSSION

Neighborhood-level inequality in female BC incidence was found in Tehran from 2008 to 2011. The most likely clusters of higher than expected BC were found in central, northern, and northeastern Tehran, whereas the most likely clusters of lower than expected incidence were located in southern Tehran.

Spatial analysis at finer scales can provide useful information about at-risk areas. Our results showed that the smoothed rates of BC incidence were variably distributed within specific districts; therefore, performing a spatial study at the district level would fail to identify these within-district inequalities. For example, on average, the neighborhoods in district 19 had lower rates of BC, but there were a few neighborhoods in this district with high rates of BC. Correspondingly, in general, the neighborhoods in districts 1, 2, and 3 had higher rates of BC, but there were a few neighborhoods with low rates within these districts.

While the spatial analysis of cancer measures at geographic resolutions such as the census tract and zip code has been frequently conducted in developed countries [11,12,23,24], it has not received sufficient attention in developing countries. In Iran, the spatial analysis of cancer measures using GIS and SaTScan have mainly been conducted at the level of provinces or counties [13,25,26], such that evidence about the spatial patterns of cancer measures at finer resolutions such as the neighborhood level are limited. In one study by Rohani et al. [27], it was found that the population living in districts 1, 2, 3, and 6 had the highest age-specific rates of BC incidence.

Smoothing the rates and conducting a spatial analysis with the SaTScan spatial scan statistic showed that the incidence of BC is a health problem in areas near the center and northern parts of Tehran, which correspond to wealthy areas with higher degrees of educational attainment and greater expenditures on health care activities [28]. It has been found that females living in wealthy areas had greater expenditures on health care activities such as screenings, resulting in BC being diagnosed more frequently [29]. Moreover, they have better access to cancer treatment facilities and adjuvant therapies, and likely have better survival rates [30].

Several methodological issues involving spatial analysis with SaTScan should be mentioned. It has been argued that the hierarchical approach (SaTScan default) for selecting the maximum size of clusters may lead to unnecessarily large and less informative clusters [22]. In the current study, the maximum size of the spatial clusters was based on the Gini coefficient. It has been suggested that the Gini coefficient provides more information about non-overlapping clusters, while avoiding overly large clusters with relatively small relative risks and smaller clusters with higher relative risks [22]. SaTScan allows a better understanding of spatial patterns with adjustment for covariates. Previously published studies have demonstrated that adjustments for area-based characteristics, such as census tract poverty, and individual characteristics of patients, including age, race/ethnicity, or stage at diagnosis, can change the observed pattern of clusters [12,24].

Our analysis has some advantages and limitations. The main advantage of the present study is that to our knowledge, this is the first study to explore the spatial patterns of female BC at the time of diagnosis at the neighborhood level in Iran. This type of spatial analysis at the neighborhood level can provide useful information to policy makers for the allocation of resources to truly needy areas. As expected, the raw SIRs per neighborhoods were dispersed due to extra-Poisson variability; to offset this challenge, we smoothed the raw SIRs using a BYM spatial model and the SEB method. The main objective of the BYM model is to take into account spatial autocorrelation in an efficient way, but the ability of the BYM model is limited where geographical units have different sizes and shapes [31]. One of the limitations of this study is related to how missing data may have induced bias in our results. Surveillance data, such as the data contained in cancer registries, are inevitably incomplete, and this is influenced by many factors such as sex, age, and socioeconomic status. As expected in ecological studies and spatial analysis, the ecological fallacy and the modifiable areal unit problem are potential sources of misleading interpretations. Another problem that was not accounted for in this study is the phenomenon known as the edge effect. This effect means that results for neighborhoods near administrative borders must be interpreted with caution, because, for example, the socioeconomic indicators of neighborhoods outside of the studied region may affect the characteristics of residents near the borders. Finally, the geocoding of the street addresses may have induced a degree of misclassification in the results.

In conclusion, female BC incidence was differently distributed across neighborhoods in Tehran. Higher than expected spatial clusters of BC incidence appeared in central and northern parts of Tehran, whereas areas with lower than expected incidence were located in southern Tehran. These observations of neighborhood inequality can be a basis for the allocation of resources and the implementation of preventive strategies in truly needy areas.

## Figures and Tables

**Table 1.** High- and low-risk clusters for female BC incidence using spatial scan statistics in Tehran (2008-2011)

	Optimal Gini coefficient	MSC	Clusters detected	Involved districts	At-risk population	Observed cases (O)	Expected cases (E)	Annual cases per 100,000	O/E	RR^1^	p-value
Total BC incidence	(n=3,080)										
Areas with high rates	0.47	0.04	Primary	3, 6	58,039	217	55.37	126.4	3.92	4.14	<0.001
			Secondary	3, 4	29,134	145	27.79	165.9	5.22	5.43	<0.001
			Tertiary	4, 8	45,449	161	43.36	118.1	3.71	3.86	<0.001
Areas with low			Primary	17, 18, 19	111,902	6	106.75	1.8	0.05	0.05	<0.001
rates			Secondary	16, 20	124,216	27	118.50	7.2	0.23	0.22	<0.001
			Tertiary	14	113,341	23	108.12	6.8	0.21	0.21	<0.001

BC, breast cancer; MSC, maximum size cluster; RR, relative risk.

1 Calculated as the observed cases divided by the expected cases within the cluster divided by the observed cases divided by the expected cases outside the cluster.
